# Influence of Processing Conditions on the Physicochemical Properties of a New-Type of Nutritional Drink—Millet Skim Milk Beverage

**DOI:** 10.3390/molecules24071338

**Published:** 2019-04-04

**Authors:** Minghui Pan, Yungang Cao, Xuelu Chi, Zheng Song, Nasi Ai, Baoguo Sun

**Affiliations:** 1 Beijing Advanced Innovation Center for Food Nutrition and Human Health, Beijing Engineering and Technology Research Center of Food Additives, Beijing Higher Institution Engineering Research Center of Food Additives and Ingredients, Beijing Technology & Business University, Beijing 100048, China; 1830202061@st.btbu.edu.cn (M.P.); chi_xl@163.com (X.C.); 1730202048@st.btbu.edu.cn (Z.S.); sunbg@btbu.edu.cn (B.S.); 2 School of Food and Biological Engineering, Shanxi University of Science and Technology, Xi’an 710021, China; caoyungang@sust.edu.cn

**Keywords:** millet, skim milk, beverage, physicochemical properties, SDS-PAGE

## Abstract

In this experiment, a new type of nutritional drink—millet skim milk beverage—was developed based on combining skim milk with millet and nutritional resource utilization. The effects of NaHCO_3_ concentrations in soaking water (0, 0.5 g/100 mL, and 1.0 g/100 mL) and blanching time (0, 15, and 30 min) on the physicochemical properties of millet skim milk were studied. The parameter changes caused by the above treatment were evaluated via color analysis, physicochemical analysis and sodium dodecyl sulfate polyacrylamide gel electrophoresis (SDS-PAGE). Soaking in water containing NaHCO_3_ had a significant (*p* < 0.05) effect on pH, specific gravity, viscosity, and stability. The blanching treatment had a significant (*p* < 0.05) influence on the total solids of the samples. However, blanching only slightly affected the physical properties of the samples. In addition, soaking and blanching treatments had significant (*p* < 0.05) effects on the b* value of millet skim milk beverage, whereas there was no significant (*p* > 0.05) change in L* and a*. SDS-PAGE analysis indicated that the blanching treatment had a significant (*p* < 0.05) effect on band 5 and band 6 and that the soaking treatment also had a significant effect on the bands of 6 and 7 (*p* < 0.05). By analyzing the substantial effects, we concluded that the optimum process conditions were soaking with 0.5 g/100 mL NaHCO_3_ solution and blanching for 15 min.

## 1. Introduction

Raw milk is classified as whole milk and skim milk. Raw milk whose fat content averages 3–4% fat [1] is defined as whole milk. The fat content of skim milk is less than 0.5% [2]. Based on the human pursuit of nutrition and healthy dairy products, skim milk is paid much attention because of its low fat and low-calorie contents. Products of skim milk not only provide nutrition, but also decrease the ingestion of fat and cholesterol. Thus, skim milk is suitable for people with hypertension or arteriosclerosis, the elderly, and people controlling their weight. Furthermore, normal people may prevent nutrition-based diseases initiated by high fat and high cholesterol levels [3,4].

China is an agricultural country that is abundant in whole grains with high yields, such as rice, millet, oat, maize, and beans. In recent years, diverse cereals have been processed into cereal beverages, such as sunflower, oat [5], rice [6], peanut [7], and corn beverages. In addition, cereals are also a substrate that has been used for the production of probiotic products [8]. Cereal beverages not only retain the advantages of grain nutrition but also increase the diversity of grain consumption. However, the combination of cereal drinks with milk is relatively rare. Millet, as a superior grain, contains a considerable quantity of essential amino acids, especially sulfur-containing amino acids (methionine and cysteine). The contents of these amino acids are higher in millet than in rice and wheat [9]. Health benefits such as decreasing tumor incidence, reduced blood pressure, cholesterol absorption, and prevention of cardiovascular diseases and cancer have been reported for millet. In addition, the nutritive value of millet has also been studied, including its ability to provide a variety of nutrients and antioxidants needed for human health [10,11,12]. A new style of milk beverage that is based on combining skim milk with millet and good utilization of nutritional resources has been produced. Use of this milk beverage also incorporates the health advantages of low-fat dairy products.

Studies have shown that using NaHCO_3_ when producing bean beverages can reduce beany flavor and that soaking samples in water containing NaHCO_3_ during the processing of sesame milk beverages is beneficial for removing off-flavor [13,14]. This experiment prepared millet samples by soaking, blanching, and grinding them; combining them with skim milk; and further processing them and then compared the changes in pH, protein, total solids, ash, specific gravity, viscosity, precipitation index, color, and SDS-PAGE parameters to explore the optimum processing conditions and provide a theoretical reference for further development of skim milk.

## 2. Results

### 2.1. Chemical Properties

The effects of production variables on the chemical composition of milk beverage are presented in Table 1. The protein content was almost not influenced almost by the treatment of soaking with different concentrations of NaHCO_3_. As the blanching time increased, the protein content of milk beverages showed a declining trend. Ash content had no significant change (*p* > 0.05) with blanching and slightly increased when millet was soaked in water containing NaHCO_3_. Soaking had a significant (*p* < 0.05) impact on the pH of the samples. There was also a tendency for the pH to increase and the acidity to decrease as blanching time increased. Soaking had less impact on the total solids than on the pH of the beverage, while blanching significantly (*p* < 0.05) decreased the content of total solids.

### 2.2. Physical Properties

The effects of production variables on the physical composition of milk beverage are shown in Table 2. The specific gravity of milk was significantly (*p* < 0.05) greater in the trials where millet was soaked in water containing NaHCO_3_ than in the control. There were nonsignificant (*p* > 0.05) differences in specific gravity as a result of the blanching treatment. The viscosity of milk beverage was significantly (*p* < 0.05) increased in milk made from millet soaked in water containing NaHCO_3_ compared to that from millet soaked in water not containing NaHCO_3_. No significant difference was noted in the viscosities of milk produced from millet soaked in water containing 0.5 g/100 mL and 1.0 g/100 mL NaHCO_3_. Millet was gelatinized after blanching, and as the blanching time increased, the degree of gelatinization increased. The viscosity increased in milk from blanched treatments compared with milk made without blanching. The precipitation index was lower, and the samples were more stable. The precipitation index of samples made with soaking water with 0.5 g/100 mL NaHCO_3_ was the lowest, and the stability was the greatest in milk produced under these soaking conditions. There were significant (*p* < 0.05) differences in stability between samples soaked with 0.5 g/100 mL and those soaked with 1.0 g/100 mL NaHCO_3_. The effect of blanching on stability was nonsignificant (*p* > 0.05). The stability with blanching for 15 min was better than that with blanching for 30 min.

### 2.3. Color Changes

The color values L*, a* and b* of various treatments are shown in Table 3. The color was recorded using CIE-L*a*b* uniform color space (CIE-Lab), where L* indicates lightness, a* indicates hue on a green (−) to red (+) axis, and b* indicates hue on a blue (−) to yellow (+) axis [13]. During soaking, L* decreased progressively with increasing of NaHCO_3_ concentration, which means that the lightness of the sample was reduced. There were significant (*p* < 0.05) differences in the yellow pigment content of the milk beverage treated by soaking. During the blanching processing, as the blanching time increased, the lightness of the milk beverage increased. Additionally, blanching had no significant effect on the a* value of milk beverage (*p* > 0.05). Due to the Maillard reaction occurring during blanching and the formation of corresponding products, the b* value of millet skim milk beverage decreased significantly (*p* < 0.05). 

**Figure 1:**
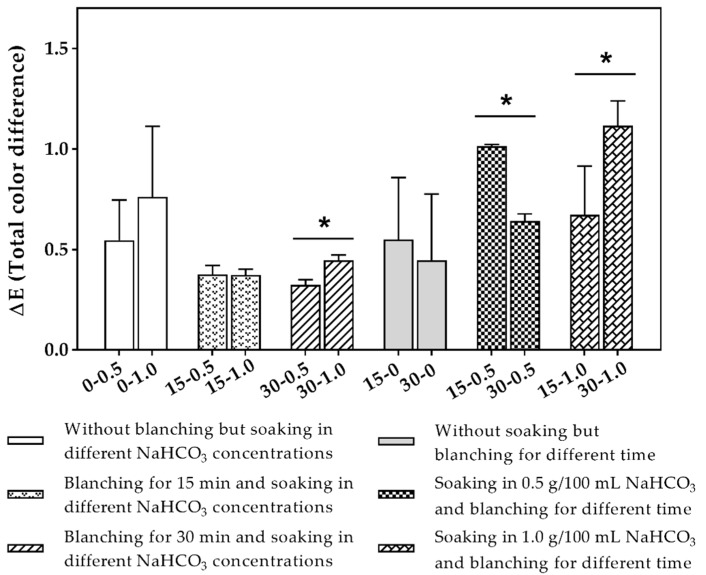
Total color difference between samples under different treatments and the corresponding control. (Labels mode of each column: blanching time-concentration of NaHCO_3_ in soaking water, e.g., 15-0.5 means blanching for 15 min and soaking in water containing 0.5 g/100 mL NaHCO_3_. * Significant (*p* < 0.05) difference between samples.).

The ∆E (total color difference) between treated samples and the corresponding control is shown as Figure 1. There was no significant (*p* < 0.05) difference between samples soaking in water containing different concentrations of NaHCO_3_ when blanched for 15 min and unblanched. However, when blanching occurred for 30 min, the differences between samples soaking in water containing 0.5 g/100 mL and 1.0 g/100 mL NaHCO_3_ were significant (*p* < 0.05). The ∆E of the 15-0.5 sample was significantly (*p* < 0.05) higher than that of the 30-0.5 sample, while that of the 15-1.0 sample was significantly (*p* < 0.05) lower than that of the 30-1.0 sample. In addition, the difference between samples that were not soaked but were blanched for different times was not significant.

### 2.4. SDS-PAGE

**Figure 2:**
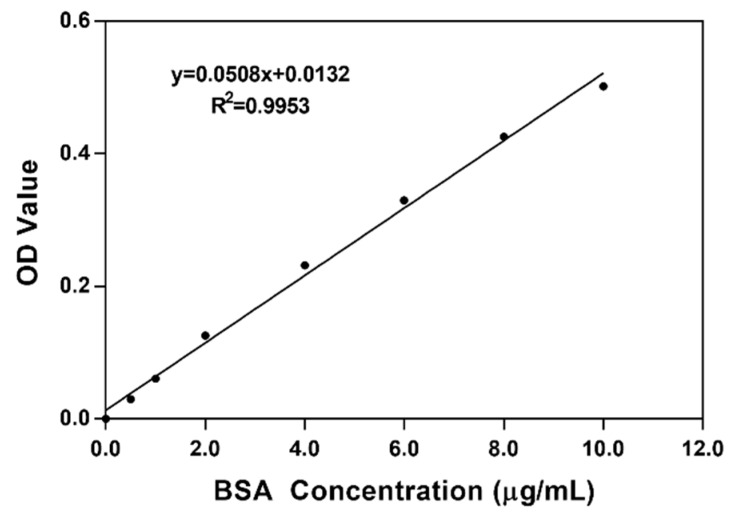
Standard curve of BSA. (OD: Optical Density; BSA: Bovine Serum Albumin).

The optimal protein injection was determined on the basis of the protein concentration standard curve shown in Figure 2. The injection volume of protein was determined after calculating the total protein content of the samples by the standard curve and the OD value of the diluted sample. Using different injection volumes for different samples ensured that the total protein contents of the samples were the same in SDS-PAGE and reduced the error caused by the difference in protein content.

**Figure 3:**
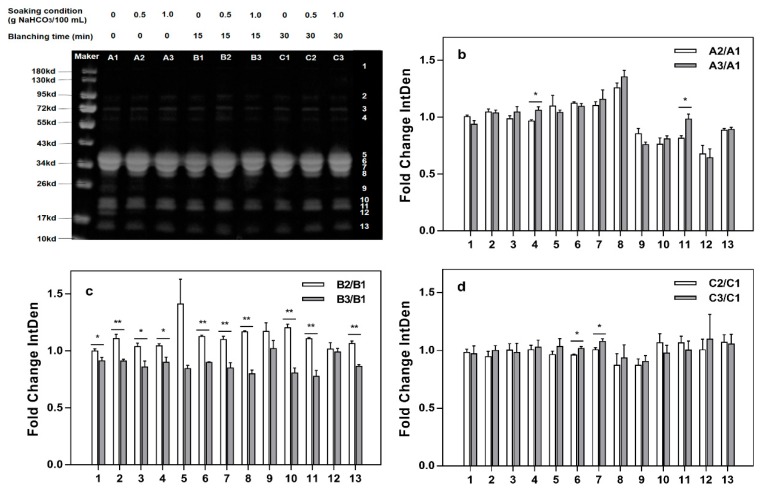
Influence of blanching on relative gray levels (**a**): sodium dodecyl sulfate polyacrylamide gel electrophoresis (SDS-PAGE) electrophoresis of the proteins from the milk beverage; (**b**): The gray level of the milk beverage produced without blanching but with soaking in water containing 0.5 g/100 mL NaHCO_3_ and 1.0 g/100 mL NaHCO_3_ relative to the gray level of the beverage without soaking and blanching; (**c**): The gray level of the milk beverage produced with blanching for 15 min and soaking in water containing 0.5 g/100 mL NaHCO_3_ and 1.0 g/100 mL NaHCO_3_ relative to the gray level of the beverage with blanching for 15 min and without soaking; (**d**): The gray level of the milk beverage produced with blanching for 30 min and soaking in water containing 0.5 g/100 mL NaHCO_3_ and 1.0 g/100 mL NaHCO_3_ relative to the gray level of the beverage with blanching for 30 min and without soaking. * Significant (*p* < 0.05) difference between samples; ** Significant (*p* < 0.01) difference between samples.

**Figure 4:**
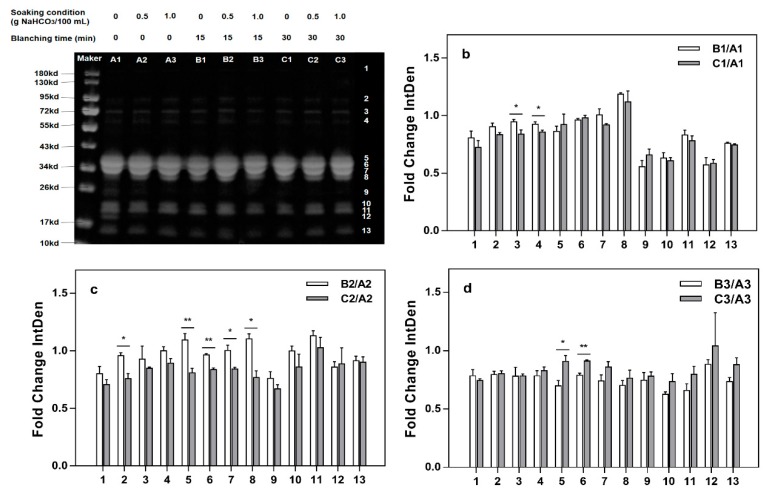
Influence of soaking (NaHCO_3_) on relative gray levels (**a**): SDS-PAGE electrophoresis of the proteins from the milk beverage; (**b**): The gray levels of the milk beverage without soaking but with blanching for 15 min and 30 min relative to that of the beverage with neither soaking nor blanching; (**c**): The gray level of the milk beverage with soaking in water containing 0.5 g/100 mL NaHCO_3_ and blanching for 15 min and 30 min, relative to that of the beverage with soaking in water containing 0.5 g/100 mL NaHCO_3_ and no blanching; (**d**): The gray level of the milk beverage with soaking in water containing 1.0 g/100 mL NaHCO_3_ and blanching for 15 min and 30 min relative to that of the beverage with soaking in water containing 1.0 g/100 mL NaHCO_3_ and no blanching. * Significant (*p* < 0.05) difference between samples; ** Significant (*p* < 0.01) difference between samples.

Figure 3 depicts the analyzed SDS-PAGE patterns of protein in milk beverages produced by blanching and soaking samples in water containing NaHCO_3_. What Figure 3b–d and Figure 4b–d showed are relative grayscale analysis results (relative grayscale, i.e., gray ratio of sample group to the control group). Gray represents the chromaticity of protein bands in SDS-PAGE determined by using ImageJ.

Figure 3 shows that without blanching, the relative gray levels of bands of 4 and 11 show significant (*p* < 0.05) differences after immersion treatment with different concentrations of NaHCO_3_ and that other bands did not change significantly (*p* > 0.05). When the sample milk beverage was blanched for 15 min, the relative gray levels of the bands 2, 6, 7, 8, 10, 11, and 13 were different significantly different (*p* < 0.01) depending on whether their samples were produced with soaking with 0.5 g/100 mL or 1.0 g/100 mL NaHCO_3_, and the relative gray level differences of strips 1, 3, and 4 were significant (*p* < 0.05). Soaking with 0.5 g/100 mL NaHCO_3_ resulted in higher gray values for each strip than soaking with 1.0 g/100 mL NaHCO_3_. Bands of 6 and 7 showed significant differences (*p* < 0.05) in gray level between samples produced with blanching for 30 min that had been immersed in different concentrations of NaHCO_3_. In addition, when the sample was soaked in 0.5 g/100 mL NaHCO_3_ solution, the relative gray level was significantly reduced in samples blanched for 30 min compared those blanched for 15 min. Thus, the blanching time was selected to be 15 min as its effect was preferred.

Figure 4 illustrates that the relative gray level of the milk beverages blanched for 15 min and 30 min and not soaked with NaHCO_3_, showed significant differences (*p* < 0.05) on strips 3 and 4. In samples soaked in 0.5 g/100 mL NaHCO_3_, the blanching time had a significant (*p* < 0.05) effect on the relative gray levels of the bands 2, 7, and 8 bands, while the difference in the relative gray level between bands 5 and 6 bands was extremely significant (*p* < 0.01). In the 1.0 g/100 mL NaHCO_3_ soaking treatment, only the relative gray levels of bands 5 and 6 bands were significantly (*p* < 0.05) affected by the blanching time, and the influence on bands 6 bands was extremely significant (*p* < 0.01). 

## 3. Discussion

### 3.1. Chemical Properties

The protein content was almost not influenced by soaking with different concentrations of NaHCO_3_. Previous research on peanut milk reported that the concentration of NaHCO_3_ in soak water did not influence the protein content [7]. Blanching led to denaturation of heat-sensitive proteins, which decreased the solubility of protein in water [7].

The amount of ash slightly increased when millet was soaked in water containing NaHCO_3_. This effect is principally due to the absorption of sodium salts by the millet from soaking media, which increased the inorganic salt content but not significantly (*p* > 0.05) change [15]. There was a trend for ash content to decrease as blanching time increased for blanched samples of milk beverage, which was caused by the leaching of minerals into the blanched water in this project [16].

In contrast, soaking had a significant (*p* < 0.05) impact on the pH of the sample. The pH of milk beverage increased and the acidity decreased with increasing concentrations of NaHCO_3_ in soaking water and blanching time, respectively. The result was the same as in previous studies. Researchers reported that the pH of peanut milk increased as the concentration of NaHCO_3_ in soaking water increased to 1.0 g/100 mL and as the blanching time increased to 30 min at 100 °C [17].

‘Total solids’ is a common technical parameter in food characterization and usually refers to total compounds in the sample. Blanching treatment significantly (*p* < 0.05) decreased the content of total solids of the beverage. It has been reported that the total solids content of peanut milk decreases as blanching time increases because of denaturation and reduction in protein solubility [17].

### 3.2. Physical Properties

The specific gravity of milk beverage significantly (*p* < 0.05) increased with soaking millet in water containing NaHCO_3_ relative to soaking millet in water not containing NaHCO_3_. This increase may be partly attributed to the softness of millet tissue that resulted from soaking with NaHCO_3_, which led to homogenate being well-distributed later and a significant (*p* < 0.05) increased in the specific gravity of the sample.

For dairy products, viscosity is an important property that affects their taste and texture. Within a certain range, the greater the viscosity is, the better the fullness of dairy products in mouthfeel, which increase their popularity with consumers [18]. The viscosity of milk beverage significantly (*p* < 0.05) increased when it is made from millet soaked in water containing NaHCO_3_ compared to millet soaked in water not containing NaHCO_3_. No significant difference was noted in the viscosities of milk produced from millet soaked in water containing 0.5 and 1.0 g/100 mL NaHCO_3_. Blanching treatments did not significantly affect viscosity. Researchers reported that heat treatment significantly affected viscosity [19]. This result may partly be attributed to the slight increase in protein solubility and the release of endogenous nutritional material in the samples soaked in 0.5 g/100 mL NaHCO_3_ compared to the control. Similar results were observed in other studies, i.e., the viscosity of soymilk significantly increased [20]. At the same time, the millet structure was effectively softened to make the homogenization sufficient with the soaking treatment. With increasing specific gravity, collisions between protein micelles increased in frequency, and the degree of cross-linking between the micelles was also increased, thereby significantly (*p* < 0.05) increasing the viscosity of the samples.

Stability is an important indicator for testing the quality of a sample. The lower the precipitation index is, the higher the stability of the sample. For milk-containing beverages, viscosity is often closely related to stability. In general, the higher the viscosity is, the tighter the cross-linking between proteins, the more homogeneous the system, and the higher the stability of the corresponding sample. Above all, the precipitation index of samples produced with soaking water containing 0.5 g/100 mL NaHCO_3_ was the lowest, and the stability of these was the greatest. In addition, the pH and protein solubility of protein will also have a certain impact on the stability of the product system [21]. There were significant (*p* < 0.05) differences in stability between samples soaked with 0.5 g/100 mL and those soaked with 1.0 g/100 mL NaHCO_3_. This result may partly be attributed to the influence of the soaking water containing 1.0 g/100 mL NaHCO_3_ on the cross-linking degree of proteins and on stabilizer function, which decreased the stability of the sample system. The effect of blanching on stability was nonsignificant (*p* > 0.05). The stability of samples produced with blanching for 15 min was greater than that with blanching for 30 min. This difference might be because blanching for a long time made the outer fiber structure of the millet change, resulting in instability. Considering all physical properties measured, the use of soak water containing 0.5 g/100 mL NaHCO_3_ and blanching for 15 min were considered as the most effective combination of treatment conditions.

### 3.3. Color Changes

The millet structure might have softened because of the NaHCO_3_ soaking treatment, which would have helped the millet contents to leave the millet and increased the fullness of the milk beverage. The yellow pigment is a unique functional ingredient of millet and mainly a carotenoid analogue that plays an important role in visual health care and treatment. There were significant (*p* < 0.05) differences in the yellow pigment content of the milk beverage treated by soaking under different conditions, but the effect was not definite. The millet homogenate was improved, and the yellowness was slightly increased, most likely because of the soaking treatment with a low concentration of NaHCO_3_. The treatment with a high concentration of the alkaline compounds NaHCO_3_ caused partial yellow pigment degradation. During blanching processing, the lightness of the milk beverage increased with increased blanching time. This change might occur because the blanching process gelatinized the starch inside the millet. It has been reported elsewhere that lightness of sesame milk increases with blanching of sesame seeds [13]. The Maillard reaction is a kind of nonenzymatic browning that is widely found in the food industry. It is a chemical reaction involving the reduction of macromolecules such as proteins and amino acids to form brown or even black macromolecular substances [22]. Due to the Maillard reaction occurring during blanching and the formation of corresponding products, the b* value of milk beverages decreased significantly (*p* < 0.05). The overall differences in ∆E indicated that all treated samples had different colors than the corresponding controls.

### 3.4. SDS-PAGE

The proteins in millet are mainly albumin/globulin, prolamin and gluten, of which alcohol-soluble prolamin (setarin) is the main protein [23]. The albumin and globulin mainly have low molecular mass, and there are obvious bands around at approximately 54 kDa, 34 kDa, and 12 kDa [24]. The prolamin mass is mainly distributed at approximately 21.7 kDa, 13.4 kDa, and 27 kDa. This is similar to the report that prolamins were distributed within the molecular weight range of 13–27 kDa. Gluten has multiple bands in both high and low molecular mass ranges [24]. In addition, the main proteins in milk are four caseins, lactoglobulin and whey protein [25]; the SDS-PAGE bands 5, 6, 7, and 8 are mainly αs_2_-casein, αs_1_-casein, β-casein, and κ-casein protein. αs_2_-Dimer is mainly distributed at approximately 94.7 kDa. α-Lactoglobulin is mainly distributed at approximately 18.4 kDa, and α-lactalbumin is mainly distributed at approximately 14.2 kDa [26,27].

According to Figure 3, the relative B2/B1 gray ratio was greater than 1 among the 13 bands, which indicated that compared to the control treatment, soaking in water containing 0.5 g/100 mL NaHCO_3_ was significantly (*p* < 0.05) conducive to protein dissolution. The relative gray level of each strip of the sample soaked in 0.5 g/100 mL is higher than that of the sample soaked in 1.0 g/100 mL. This result indicated that the 0.5 g/100 mL NaHCO_3_ soaking treatment could release the protein in millet more effectively after blanching for 15 min than without blanching. After the samples were blanched for 30 min, bands 6 and 7 showed significant differences (*p* < 0.05) in relative gray levels for samples produced using different concentrations of NaHCO_3_. In addition, when the sample was soaked in 0.5 g/100 mL NaHCO_3_ solution, the resulting relative gray level was significantly reduced when with blanching for 30 min compared with blanching for 15 min. Thus, the blanching time was selected to be 15 min.

Figure 4 shows that the relative gray level of the milk beverage, which was produced with blanching for 15 min and 30 min and with soaking in water without NaHCO_3_ showed significant differences (*p* < 0.05) on strips 3 and 4. With soaking with 0.5 g/100 mL NaHCO_3_, the difference in the relative gray level between bands 5 and 6 was extremely significant (*p* < 0.01). Strips 5 and 6, in addition to showing casein in skim milk, included albumin in millet. Scientific studies have shown that temperature and moisture changes have different effects on the solubility of proteins in millet. The water-soluble, salt-soluble, alcohol-soluble protein contents in millet increased during the appropriate heat treatment. When blanched for 30 min, the water content in the system was reduced, thus, the solubility of the millet protein was lowered, which indicated that with the 0.5 g/100 mL NaHCO_3_ soaking treatment, the effect of blanching for 15 min was obviously better than that of blanching for 30 min [28]. In the 1.0 g/100 mL NaHCO_3_ soaking treatment, only the relative gray levels of bands 5 and 6 were significantly (*p* < 0.05) affected by the blanching time, and the influence on bands 6 was extremely significant (*p* < 0.01). However, as shown in Figure 4d, the relative gray B3/A3 and C3/A3 ratios were both less than 1, which indicated that the 1.0 g/100 mL NaHCO_3_ soaking treatment was not conducive to protein dissolution in millet. Therefore, the 0.5 g/100 mL NaHCO_3_ soaking treatment was effective in this processing.

From the SDS-PAGE results, it could be concluded that blanching for 15 min and soaking with 0.5 g/100 mL NaHCO_3_ was the best combination of processing methods.

## 4. Materials and Methods 

### 4.1. Materials

Skim milk was obtained from the Beijing Sanyuan Food Corp., Ltd., (Beijing, China) and putted in a light resistant and refrigerated container. The sample was carried back to laboratory within two hours. Using a MilkoScan^TM^ Minor milk analyzer, we determined the fat, protein and lactose contents of the milk. The results showed that fat content was less than 0.5% (*w*/*w*), the protein content was 3.17% (*w*/*w*) and the lactose content was 3.77% (*w*/*w*). After the test, milk was stored at 4 ± 1 °C for subsequent applications. Millet was obtained from the local market.

Xanthan, carboxymethyl cellulose (CMC), concentrated sulfuric acid, NaOH, HCl, Na_2_CO_3_, boric acid, methyl-red, and bromocresol green were obtained from J&K Chemicals. Food-grade baking soda (NaHCO_3_) was obtained from the local market.

### 4.2. Methods

#### 4.2.1. Preparation of Samples

**Figure 5:**
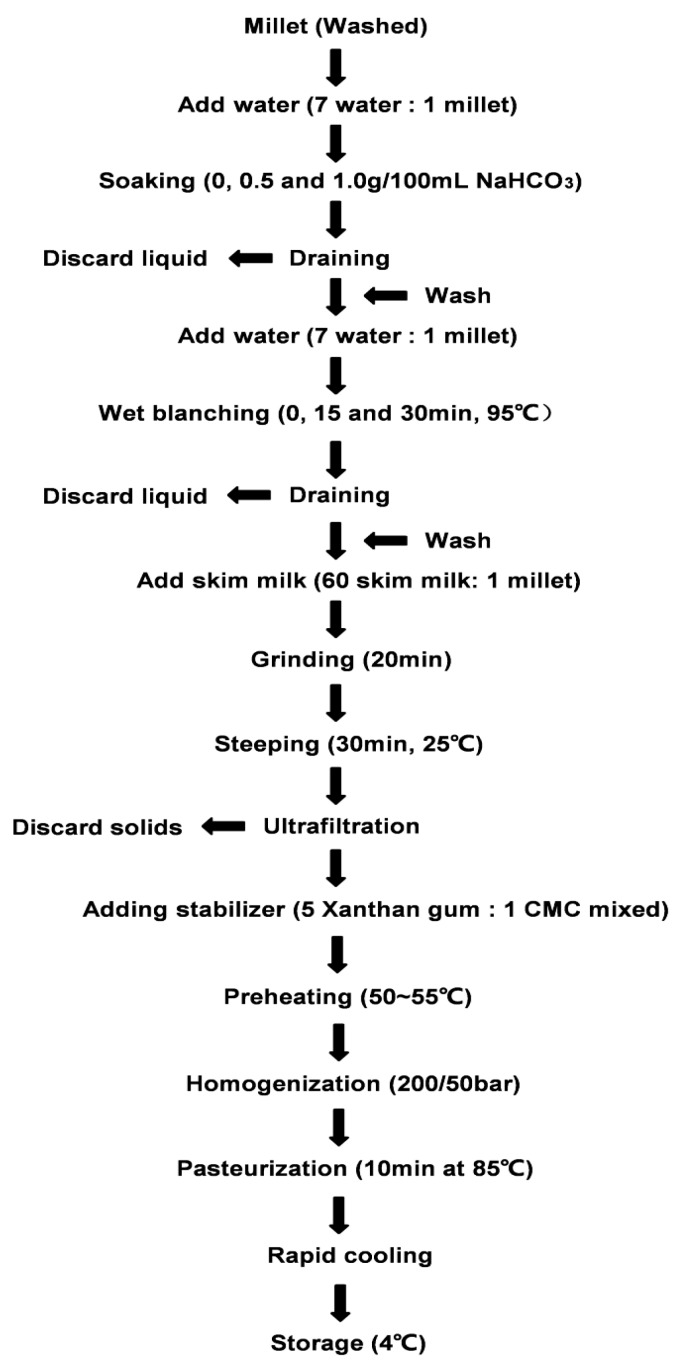
Flow diagram for the preparation of millet skim milk beverage.

The technological process for making millet skim milk beverage is shown in Figure 5. Operating points of the process included: (a) cleansing of millet (removed impurities of millet and production without dust or any other granules), and (b) soaking with NaHCO_3_ (the ratio of the NaHCO_3_ solution to millet was 7:1, with concentration of 0, 0.5 g/100 mL, and 1.0 g/100 mL). After 16 h of soaking time, the NaHCO_3_ solution was removed. (c) For a wet blanching step, millet was mixed with water (water: millet, 7:1) and blanched at 95 °C for 0, 15 min, and 30 min and then refrigerated. (d) The sample was ground with skim milk (60 skim milk:1 millet) at 9000 rpm for 20 min [15]. (e) For ultrafiltration, rough filtration was performed with absorbent gauze and subsequent vacuum filtration (*p* < 0.6 MPa). (f) Stabilizer was then added in the following manner. A rotor was placed in the milk beverage after filtration, and the whole beverage was placed in a water bath at 40 °C. Stabilizer was added (xanthan gum 0.05 g/100 mL and CMC 0.01 g/100 mL), and the samples was heated to 85 °C step by step and held there for 15 min. (g) For homogenization, pasteurization and storage, the milk beverage was then homogenized twice at 60 °C and a pressure of 200/50 bar. The homogenized milk beverage was pasteurized at 85 °C for 10 min, cooled rapidly and then stored at 4 °C before further analysis.

#### 4.2.2. Physical and Chemical Analysis

The pH of the milk beverage was measured with a digital pH meter (InsMark Instrument, Shanghai, China) at 25 °C. Protein, ash, total solids and specific gravity were analyzed by the reference Association of Official Analytical Chemists (AOAC) method [29].

#### 4.2.3. Precipitation Index

The milk beverages were centrifuged in a centrifuge (CR22N, Hitachi, Japan) at 2500× *g* for 10 min, and then the supernatant was removed by placing the tubes upside down for half a minute. The resulting precipitate was weighed to calculate precipitation as a fresh weight percentage [13].

#### 4.2.4. Viscosity

The viscosity of milk beverage was determined at 25 °C by a rotational viscometer (DV-III+ULTRA, Brookfield, MA, USA) with a rotor size of 31 and a speed of 5 rpm.

#### 4.2.5. Color Changes

Color properties play an important role in visual impressions [30]. The changes in the coloration of milk beverage were measured by using a USPRO Colorimeter (USPRO Colorimeter CM-3500d, Minolta, Knoica, Japan). A blackboard calibration was performed with the destination mask of φ30mmCM-A123. A whiteboard calibration was completed with the destination mask of φ8mmCM-A122 together with a quartz cell CM-A97 (2 mm) filled with 7 mL of deionized water. The color was recorded using the CIE-L*a*b* uniform color space (CIE-Lab) where L* indicates lightness, a* indicates hue on a green (−) to red (+) axis, and b* indicates hue on a blue (−) to yellow (+) axis. Then the samples were analyzed in three parallels experiments. 

The ∆E was calculated between samples with different treatments and the corresponding control (designated with the index 0) with fixed one factor [31]:

(1) $$\Delta E=\sqrt{{\left(L_{0}{}^{*}-L^{*}\right)}^{2}\;+{\left(L_{0}{}^{*}-a^{*}\right)}^{2}+\;{\left(b_{0}{}^{*}-b^{*}\right)}^{2}}$$

#### 4.2.6. SDS-PAGE

BCA standard curve: A standard curve was constructed with bicinchoninic acid (BCA) working solution that was made of 50 parts BCA solution A together with 1 part BCA solution B. Then, 10 μL of standard protein was diluted with PBS to 100 μL for standby application. Then, 0, 0.5, 1, 2, 4, 6, 8, and 10 μL of standard products were added to a 96-well plate and increased to 20 μL volumes with PBS. BCA working solution was added to each hole and then kept in 37 °C for 30 min. The standard curve was plotted using OD values that were measured by a microplate reader (Full-function micro orifice plate detector SynergyH1, BioTek, Winooski, VT, USA) [32].

Calculation of protein concentration: Samples to be tested were diluted 20 times by using PBS solution, and then, 10 μL of the diluted sample was added to a 96-well plate together with 200 μL of BCA working solution. OD values were measured by a microplate reader, and each sample was analyzed with three parallels experiments. According to the standard curve and OD values of samples, the protein concentration was calculated precisely.

SDS-PAGE: The optimum amount of protein to be injected was confirmed by the protein content. Sodium dodecyl sulfate polyacrylamide gel electrophoresis (SDS-PAGE) was carried out according to the method of the study by Laemmli [33], using a 12% resolving gel and a 4% stacking gel combined with 80 V constant voltage electrophoresis for 6 h. After the treatment, the gel tablet was dyed by CBB R-250 for 4 h and decolored with acetic acid (volume ratio of 3:1) until a hyaline appearance was reached. The gel tablet was imaged by the UVP Vilber Lourmat and combined with ImageJ to analyze grayscale levels of each lane protein.

#### 4.2.7. Statistical Analysis

The histogram in the experiment was generated by GraphPad Prism 7 (GraphPad Software, Inc., La Jolla, CA, USA), and the grayscale levels of each SDS-PAGE lane were analyzed by ImageJ. All experimental data were processed with one factor analysis of variance (ANOVA) using SPSS 23 (IBM Deutschland GmbH, Ehningen, Böblingen. Germany); *p* < 0.05 indicates that differences are significant.

## 5. Conclusions

This study was conducted to analyze the physical properties, chemical properties, color changes, and SDS-PAGE properties of different processed millet skim milk beverages. The test showed that soaking in water containing NaHCO_3_ reduced the protein content in the sample, the precipitation index of stability and the color L* and a* values, but the percentage of the ash content, pH, specific gravity and viscosity increased. The blanching treatment reduced the content of protein, ash, total solids and color a* and b* values and increased the specific gravity, viscosity, precipitation index of stability, and L* value of the color. Viscosity and stability are important indicators for sample quality inspection. Soaking in water containing 0.5 g/100 mL NaHCO_3_ significantly (*p* < 0.05) increased the viscosity and stability of the product while increasing the lightness of the product. Compared with the control treatment and soaking with 1.0 g/100 mL, soaking in water containing 0.5 g/100 mL was better. Blanching for 15 min effectively increased the viscosity of the product compared with the control product, and the products were lighter in the former case. Compared with blanching for 30 min, short-term blanching treatment improved the stability of the millet skim milk beverage sample system. In addition, for economic reasons, blanching for 15 min was the best choice. Based on the parameter changes caused by the two treatment methods, soaking with 0.5 g/100 mL NaHCO_3_ solution and blanching for 15 min was the best production process.

The development of skim milk has been limited due to its mouthfeel defects, and the novel beverage produced by combining with skim milk and millet is a new beginning to improve the skim milk processing, but it is only a small step in this development. As a light food drink in modern life, skim milk is attracting increasing attention from scientific research and consumers. It is believed that in the constant exploration of researchers and the unremitting pursuit of consumers, skim milk products will lead to a new trend in light food.

## Figures and Tables

**Table 1:** Chemical properties of millet milk influenced by production variables.

Treatment	Level	Protein (g/100 g)	Ash (g/100 g)	pH	Total Solids (g/100 g)
Soaking conditions (g NaHCO_3_/100 mL)	0	3.26 ± 0.19 ^a^	6.65 ± 0.027 ^a^	6.65 ± 0.027 ^b^	11.17 ± 0.33 ^a^
	0.5	3.11 ± 0.16 ^a^	6.79 ± 0.030 ^a^	6.69 ± 0.000 ^ab^	10.17 ± 0.44 ^a^
	1.0	3.20 ± 0.08 ^a^	6.73 ± 0.071 ^a^	6.73 ± 0.003 ^a^	11.17 ± 0.44 ^a^
Blanching time (min)	0	3.26 ± 0.19 ^a^	6.65 ± 0.027 ^a^	6.65 ± 0.046 ^b^	11.17 ± 0.33 ^a^
	15	3.20 ± 0.15 ^a^	6.50 ± 0.089 ^a^	6.69 ± 0.003 ^ab^	10.00 ± 0.29 ^b^
	30	3.02 ± 0.03 ^a^	6.58 ± 0.021 ^a^	6.72 ± 0.006 ^a^	9.33 ± 0.33 ^b^

^a,b^ Significant (*p* < 0.05) difference between samples. Values within the same treatment but with different levels followed by the same letter were not significantly different at the 5% level.

**Table 2:** Physical properties of millet milk influenced by production variables.

Treatment	Level	Specific Gravity	Viscosity (cP)	Precipitation Index (%)
Soaking conditions (g NaHCO_3_/100 mL)	0	1.031 ± 0.0002 ^b^	9.03 ± 0.28 ^b^	2.30 ± 0.09 ^b^
	0.5	1.033 ± 0.0003 ^a^	12.72 ± 1.35 ^a^	1.73 ± 0.04 ^b^
	1.0	1.034 ± 0.0003 ^a^	11.41 ± 0.38 ^ab^	5.14 ± 0.32 ^a^
Blanching time (min)	0	1.031 ± 0.0002 ^a^	9.03 ± 0.28 ^a^	2.30 ± 0.09 ^a^
	15	1.033 ± 0.0014 ^a^	9.10 ± 0.67 ^a^	2.29 ± 0.40 ^a^
	30	1.032 ± 0.0003 ^a^	9.31 ± 1.14 ^a^	2.43 ± 0.34 ^a^

^a,b^ Significant (*p* < 0.05) difference between samples. Values within the same treatment but with different levels followed by the same letter were not significantly different at the 5% level.

**Table 3:** Color analysis of millet milk influenced by production variables.

Treatment	Level	L*	a*	b*
Soaking conditions (g NaHCO_3_/100 mL)	0	67.67 ± 0.32 ^a^	1.95 ± 0.06 ^a^	17.51 ± 0.13 ^b^
	0.5	67.50 ± 0.05 ^a^	1.91 ± 0.08 ^a^	17.83 ± 0.02 ^a^
	1.0	67.03 ± 0.08 ^a^	1.82 ± 0.01 ^a^	17.37 ± 0.01 ^b^
Blanching time (min)	0	67.67 ± 0.32 ^a^	1.95 ± 0.06 ^a^	17.51 ± 0.13 ^a^
	15	67.78 ± 0.04 ^a^	1.86 ± 0.009 ^a^	17.18 ± 0.02 ^b^
	30	67.84 ± 0.04 ^a^	1.87 ± 0.007 ^a^	17.31 ± 0.04 ^ab^

^a,b^ Significant (*p* < 0.05) difference between samples. Values within the same treatment but with different levels followed by the same letter were not significantly different at the 5% level.
